# Localization and phosphorylation of *Plasmodium falciparum* nicotinamide/nicotinate mononucleotide adenylyltransferase *(PfNMNAT)* in intraerythrocytic stages

**DOI:** 10.1186/s12936-018-2307-4

**Published:** 2018-04-11

**Authors:** Carlos A. Nieto, Lina M. Sánchez, Diana M. Sánchez, Gonzalo J. Díaz, María H. Ramírez

**Affiliations:** 1 Laboratorio de Investigaciones Básicas en Bioquímica (LIBBIQ), Facultad de Ciencias, Universidad Nacional de Colombia, Sede Bogotá, Bogotá D.C., Colombia; 2 Laboratorio de Toxicología, Facultad de Medicina Veterinaria y Zootecnia, Universidad Nacional de Colombia, Sede Bogotá, Bogotá D.C., Colombia

**Keywords:** *Plasmodium falciparum*, NAD+ metabolism, Malaria, PfNMNAT, Immunolocalization, Phosphorylation

## Abstract

**Background:**

Nicotinamide adenine dinucleotide (NAD+) is an essential molecule in the energy metabolism of living beings, and it has various cellular functions. The main enzyme in the biosynthesis of this nucleotide is nicotinamide/nicotinate mononucleotide adenylyltransferase (NMNAT, EC 2.7.7.1/18) because it is the convergence point for all known biosynthetic pathways. NMNATs have divergences in both the number of isoforms detected and their distribution, depending on the organism.

**Methods:**

In the laboratory of basic research in biochemistry (LIBBIQ: acronym in Spanish) the NMNATs of protozoan parasites (*Leishmania braziliensis*, *Plasmodium falciparum*, *Trypanosoma cruzi*, and *Giardia duodenalis*) have been studied, analysing their catalytic properties through the use of proteins. Recombinants and their cellular distribution essentially. In 2014, O’Hara et al. determined the cytoplasmic localization of NMNAT of *P. falciparum*, using a transgene coupled to GFP, however, the addition of labels to the study protein can modify several of its characteristics, including its sub-cellular localization.

**Results:**

This study confirms the cytoplasmic localization of this protein in the parasite through recognition of the endogenous protein in the different stages of the asexual life cycle. Additionally, the study found that PfNMNAT could be a phosphorylation target at serine, tyrosine and threonine residues, and it shows variations during the asexual life cycle.

**Conclusions:**

These experiments confirmed that the parasite is situated in the cytoplasm, fulfilling the required functions of NAD+ in this compartment, the PfNMNAT is regulated in post-transcription processes, and can be regulated by phosphorylation in its residues.

**Electronic supplementary material:**

The online version of this article (10.1186/s12936-018-2307-4) contains supplementary material, which is available to authorized users.

## Background

*Plasmodium falciparum* causes malaria and is the leading parasitic cause of death worldwide [1]. Given that, to date, there is no clinically available vaccine and considering the recent increase in drug-resistant parasite strains, it is necessary to find proteins that serve as therapeutic targets for control of the disease [2].

During its asexual life cycle, the parasite infects erythrocytes, increasing oxidative stress intracellular after haemoglobin degradation. For this reason, the parasite has a variety of antioxidant systems that depend directly on the reducing power of the cellular content of NADPH. In addition to infecting the erythrocyte, *P. falciparum* increases the activity of the pentose phosphate cycle, NAD+ synthesis, and the expression of glycolytic enzymes in order to adapt to the intracellular environment [3, 4]. Therefore, the role of NAD(P)+ is essential since it is a key factor in some essential biological and biochemical processes of the parasite [5]. That is why the identification and characterization of the enzymes involved in its biosynthesis prove interesting. One of the most important enzymes in this pathway is the nicotinamide/nicotinate mononucleotide adenylyltransferase (NMNAT; EC:2.7.7.1) as it is the point of convergence of the two NAD+ biosynthetic pathways, de novo and salvage [6]. NMNAT has been identified in organisms as diverse as archaea, bacteria and eukaryotes [7, 8]. In humans, three isoforms (NMNATs 1, 2 and 3) have been identified in the nucleus, Golgi apparatus and mitochondria, respectively [9, 10]. Each of them has specific sequences that allow its intracellular localization.

*Plasmodium falciparum* NMNAT has already been identified [11, 12], and its tertiary structure has also been recently resolved by X-ray analysis [13]. This article describes the sub-cellular localization, phosphorylation and variations of NMNAT during the asexual life cycle of the parasite.

## Methods

### Synthesis and evaluation of polyclonal anti-His-PfNMNAT antibodies

IgG antibodies were obtained using the previously standardized protocol [14], in which 50 μg of recombinant protein previously obtained in another job [11] was used to perform 4 inoculations in 6-week-old BALB-C mice. Blood collection was performed every 8 days after inoculations. Antibodies were purified using affinity chromatography [15]. To obtain IgY, 19-week old chickens (Babcock Brown) were inoculated 4 times with 150 μg of the recombinant protein [16]. Eggs and blood were collected from day 0 to 1 month after the last inoculation. Antibodies were purified from egg yolk by thiophilic resin chromatography. For the evaluation of antibodies, different concentrations of recombinant His-PfNMNAT (3–125 ng) were separated by SDS-PAGE, electroblotted onto a PVDF membrane and developed with HRP. As a primary antibody, the sera used were obtained from avian (blood and egg yolk) and murine models at a dilution of 1:5000. As a control, 125 ng of BSA was used.

### *Plasmodium falciparum* culture

FCR-3 strains of *P. falciparum* were cultured in vitro [17]. The parasites were synchronized with 5% sorbitol at 37 °C for 10 min [18]. Synchronic cultures in the ring stage were maintained in culture until reaching the trophozoite and schizont stages. Parasites at different asexual stages were obtained by centrifugation at 4000×*g* for 5 min, and erythrocytes were lysed with 0.01% saponin in PBS buffer at 4 °C for 15 min. The parasites were recovered by centrifugation at 17,000×*g* for 15 min, and then they were washed with 1× PBS until complete removal of the erythrocyte membrane and haemoglobin residues.

### Collection of the cytoplasmic protein extracts

Approximately 2–4 million parasites were resuspended in 100 μl of Tris–magnesium gelatin (TMG) buffer (10 mM Tris–HCl, pH = 7.5, 1.5 mM MgCl_2_, 10 mM B-mercaptoethanol, and 10% glycerol) and protease inhibitor (Sigma, P8340; 1 mM AEBSF, 14 μM E64, 15 μM pepstatin A, 40 μM bestatin, 20 μM leupeptin, and 0.8 μM aprotinin). The extracts were then lysed by incubation with 0.2% NP-40 at 4 °C for 30 min and sonicated for 30 s with 50% amplitude. Cell debris was removed by centrifugation at 17,000×*g* for 30 min at 4 °C.

### Immunodetection of PfNMNAT

Approximately 80–100 μg of soluble protein extract was separated on 12% SDS-PAGE gels and transferred to PVDF membranes (Thermo) for 2 h at 200 mA in electrotransfer buffer (Tris base 25 mM, 192 mM glycine and 10% V/V methanol, pH 8.3). The membranes were blocked in TBST-milk (TBST-L) for 12 h and incubated for another 12 h with the previously obtained sera (IgY or IgG) at a dilution of 1:1000 in TBST-L. Three washes were performed with TBST-L for 10 min each, before the samples were incubated for 2 h with the secondary antibody anti-mouse IgG or anti-chicken IgY coupled to biotin (1:8000) and 3 washes were made with TBST-L. The immunodetection reaction was developed with the chromogenic BCIP/NBT substrate system for streptavidin-conjugated alkaline phosphatase. The recombinant protein MBP-PfNMNAT was used as a positive control [19].

### Immunofluorescence of PfNMNAT

To determine the subcellular localization of the endogenous protein by immunofluorescence, the protocol reported by Tokin et al. [20] was followed, and the pre-immune serum of the immunized chickens was used as the negative control. The plates were mounted with 10 μl of Fluoromount (Sigma) per slide, and the images were recorded with the Nikon C1 plus confocal fluorescence microscope using a 100× objective.

### Identification of protein levels

To obtain parasites in a single stage, the culture was subjected to 3 synchronization cycles with sorbitol 5%, with pauses of 96 h (2 life cycles), between each synchronization. The parasites were obtained and lysed as mentioned above, we continued with the same immunodetection protocol where approximately 150 μg of protein was loaded for each of the stages. The antibodies used were those obtained from chicken blood. The recombinant protein MBP-PfNMNAT was used as a positive control [19].

### Immunoprecipitation of PfNMNAT

Cytoplasmic protein extracts were prepared as described above using 1 mM Na_3_VO_4_. The extract was clarified for 1 h at 4 °C with constant stirring using 100 μl of thiophilic resin (Pierce) previously equilibrated with TMG buffer. The clear extract was incubated overnight with 50 μl of purified anti-His-PfNMNAT IgY antibody from chicken serum at 4 °C with constant stirring. 100 μl of thiophilic resin previously equilibrated with TMG buffer was added, and the sample was incubated for 2 rs at 4 °C with constant stirring. The immunoprecipitate was obtained by centrifuging the samples at 4500×*g* for 3 min at 4 °C. The precipitate obtained was washed 3 times with 500 μl of TMG buffer for 10 min in each case. The final pellet was resuspended in 80 μl of loading buffer for SDS-PAGE and incubated in boiling water for 6 min. The samples obtained were analysed by silver-stained SDS-PAGE. At the same time, Western blotting was performed with anti-His-PfNMNAT antibodies and antibodies to identify phosphorylations at the S, Y and T residues (Sigma).

## Results

### Development of an immunological tool for PfNMNAT identification

**Fig. 1:**
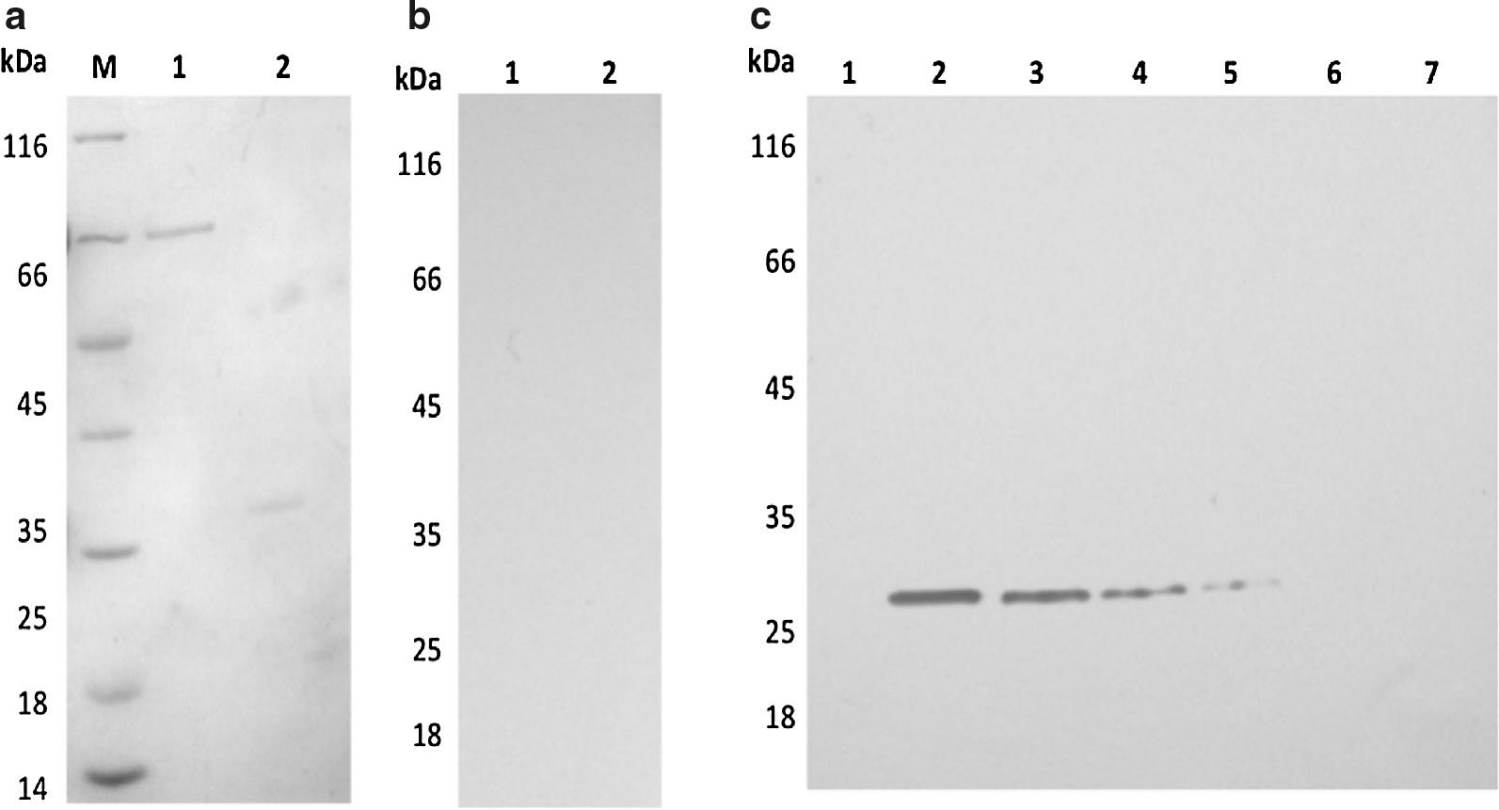
Evaluation of anti-His-PfNMNAT IgG antibodies on the recombinant protein. **a** 12% SDS-PAGE stained with Coomassie brilliant blue R-250, 1. 200 ng BSA; 2. 200 ng His-PfNMNAT; M. molecular weight marker (Thermo Scientific). **b** Western blot, polyvinylidene difluoride (PVDF) membrane, horseradish peroxidase (HRP) system. Unrelated serum murine (1:1000 dilution): 1. 125 ng BSA 2; 125 ng His-PfNMNAT. **c** Western blot, PVDF membrane, HRP system. Murine primary antibody (1:5000) for identification of recombinant His-PfNMNAT: 1. 125 ng BSA; 2–7. His-PfNMNAT: 2. 125 ng; 3. 60 ng; 4. 30 ng; 5. 15 ng; 6. 7 ng; 7. 3 ng

Avian and murine antibodies were evaluated by western blot using a recombinant protein (His-PfNMNAT). Both IgG and IgY at a dilution of 1:5000 recognize up to 15 ng of the purified recombinant protein. Figure 1 shows the evaluation of IgG, where the electrophoretic separation of His-PfNMNAT (~ 28 kDa) and bovine serum albumin (BSA, ~ 66 kDa), used as a non-specific control, can be observed (a). Antibodies specifically recognized the recombinant protein (c). Similar results were obtained with IgY (Additional files 1 and 2).

### PfNMNAT is identified in the soluble extracts of the parasite

**Fig. 2:**
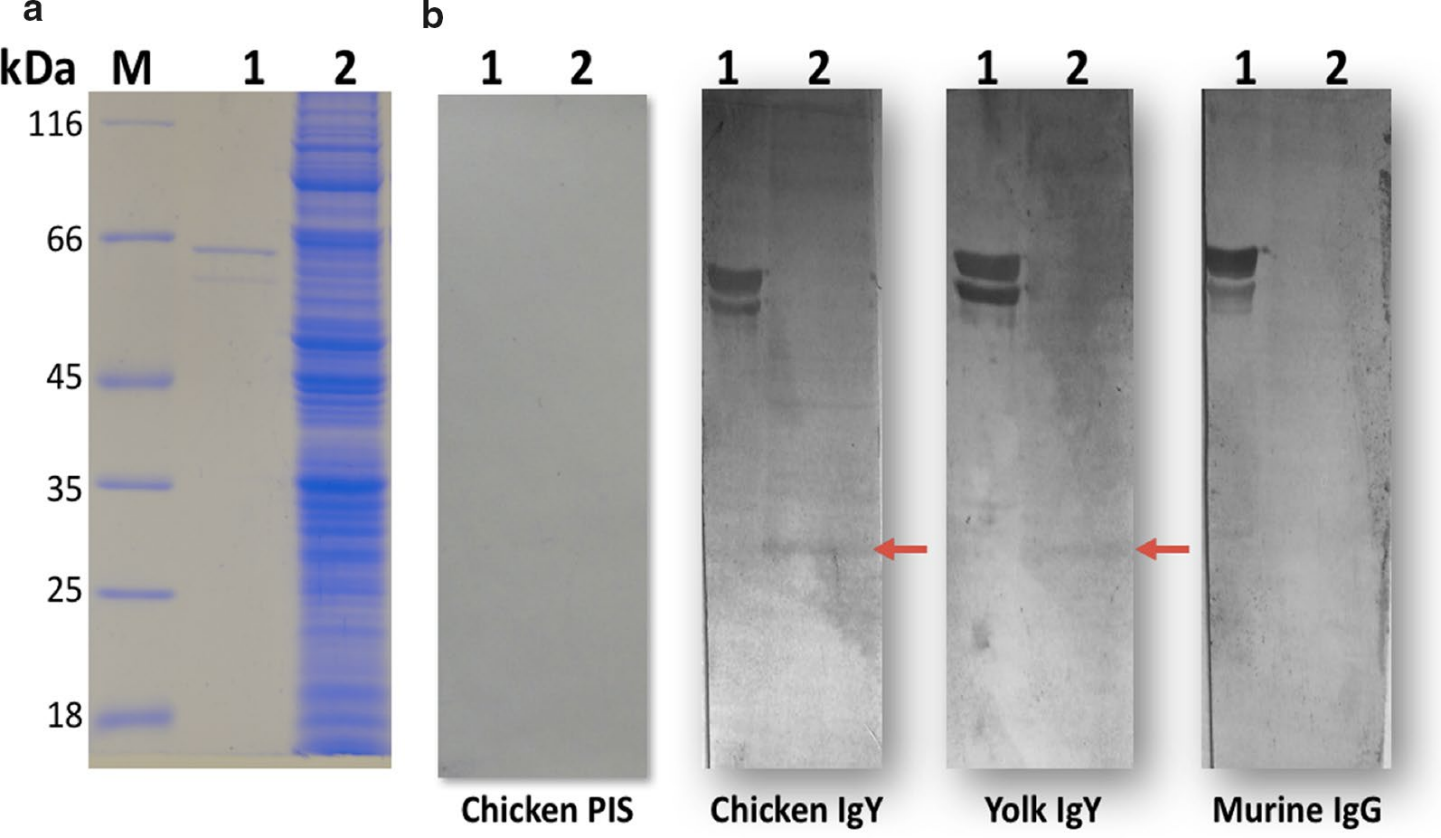
Evaluation of anti-His-PfNMNAT IgY and IgG antibodies on *Plasmodium falciparum* asynchronous extracts. **a** 12% SDS-PAGE stained with Coomassie brilliant Blue R-250. **b** PVDF membrane. 1. Positive control: Recombinant protein MBP-PfNMNAT (66 kDa). 2. 80 µg of *Plasmodium falciparum* FCR3 extract from asynchronous culture. M. molecular weight marker (Thermo Scientific). The red arrow shows the identification of a protein of approximately 28 kDa corresponding to endogenous PfNMNAT. Primary antibody 1:1000 (pre-imune serum IgY, anti-His-PfNMNAT chicken serum IgY, anti-His-PfNMNAT egg yolk IgY, or murine blood IgG), secondary antibody 1:8000 (biotinylated anti-IgY or anti-IgG), streptavidin–alkaline phosphatase 1:8000, developed with the chromogenic 5-bromo-4-chloro-3′-indolyphosphate/nitro-blue tetrazolium (BCIP/NBT) substrate system

By employing the above-mentioned antibodies on cellular extracts from asynchronous cultures of *P. falciparum*, a band of ~ 28 kDa (slightly larger than the expected theoretical mass ~ 26 kDa), was immunodetected, in addition to the identification of the control recombinant protein (MBP-PfNMNAT). When pre-immune serum was used, there was no identification, as expected (Fig. 2). The difference in mass possibly corresponds to a post-translational protein modification, taking into account what has been reported for other NMNATs. HsNMNAT-1 contains one serine residue (Ser136), which is a phosphorylation target of protein kinase C enzymes, and is involved in the regulation of the catalytic activity of the self-modified PARP1 enzyme [21]. It has recently been found that *Trypanosoma cruzi* NMNAT has one or more serine-phosphorylated residues [22], which are possibly necessary for its regulation. PfNMNAT may be regulated by phosphorylation in some of its residues.

### PfNMNAT is a phosphorylation target

**Fig. 3:**
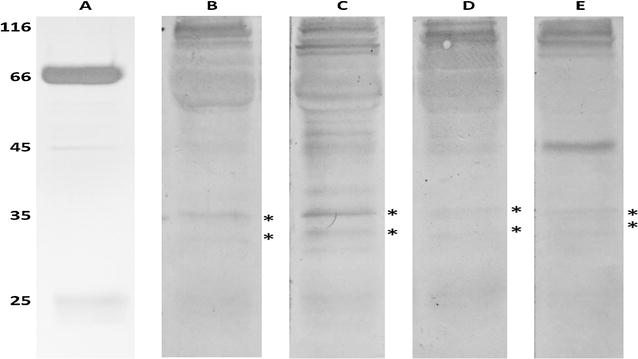
PfNMNAT phosphorylation. PfNMNAT was immunoprecipitated using IgY antibodies obtained from chicken serum and thiophilic resin. A–E 15 μl of the immunoprecipitated product A. 12% SDS-PAGE silver-stained. B–E PVDF membrane. B Primary antibody 1:1000 anti-His-PfNMNAT IgG. C Anti-T-phosphorylated IgG. D Anti-Y-phosphorylated IgG. E Anti-S-phosphorylated IgG. Secondary antibody 1:8000 (biotinylated anti-IgY or anti-IgG), streptavidin–alkaline phosphatase 1:8000, developed with the BCIP/NBT chromogenic substrate system

The NetPhos 3.1 server [23] predicted 21 possible phosphorylation sites for PfNMNAT: 15 for serine, 2 for tyrosine and 4 for threonine. To determine whether PfNMNAT is a phosphorylation target at some of its residues, protein immunoprecipitation was performed in the soluble extract of the parasite using both murine and chicken serum antibodies. Immunoprecipitated proteins were subjected to Western blot analysis with anti-phosphorylated S, Y and T antibodies, as described in the methodology. In both experiments, the results were congruent, finding that PfNMNAT is phosphorylated at one or more serine, tyrosine and threonine residues (Fig. 3). Pease et al., by means of a phosphoproteome analysis of the parasite using mass spectrometry, found that the residue of serine 167 is a phosphorylation target and has a maximum peak of modification in the ring stage [24]. This corroborates the results obtained and could be an explanation for the increase in protein size displayed in the Western blot analysis.

### PfNMNAT is located in the cytoplasm of the parasite

**Fig. 4:**
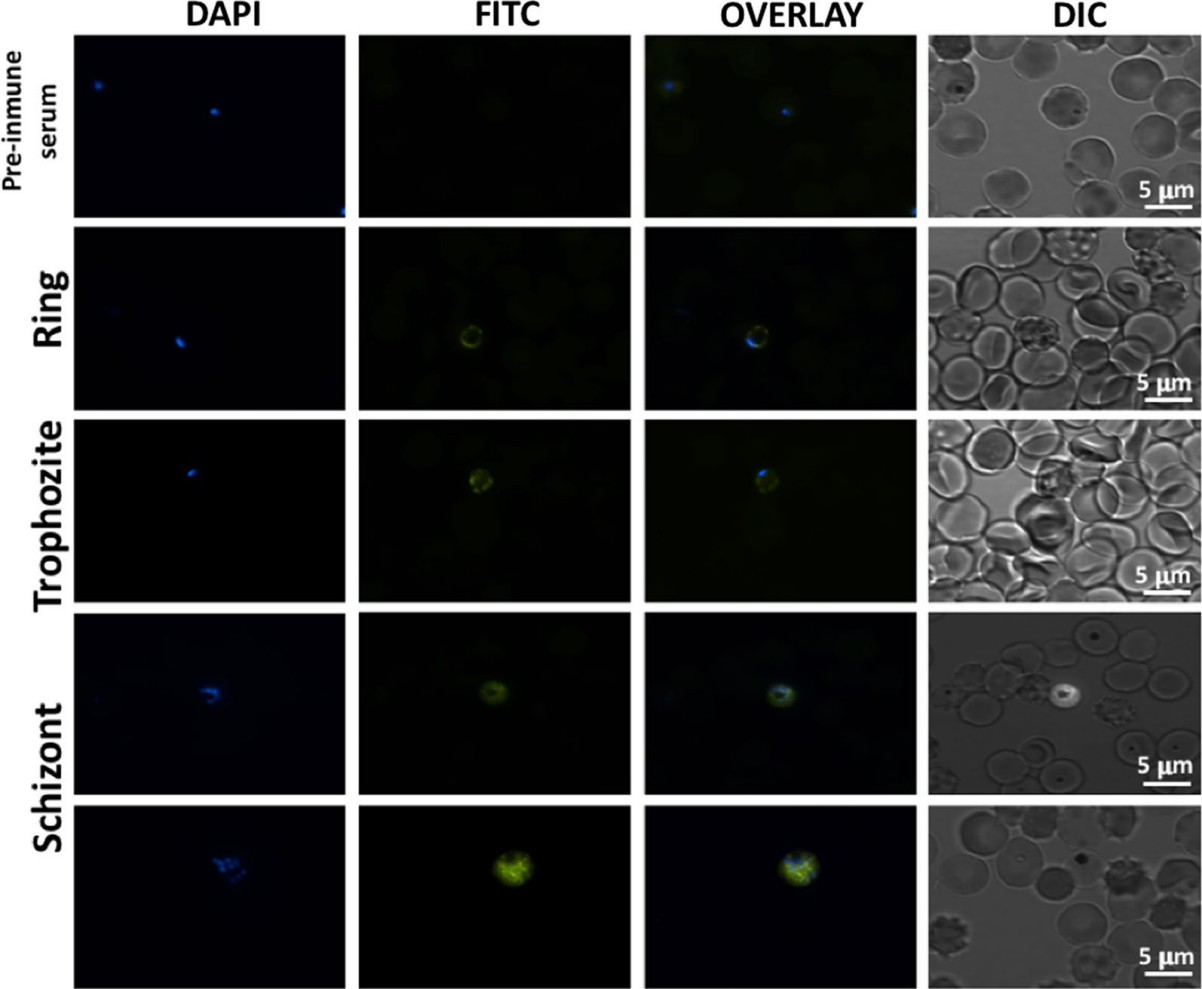
Immunolocalization of PfNMNAT at different stages of the asexual life cycle of *Plasmodium falciparum.* Primary antibody: anti-His-PfNMNAT IgY serum (1:250). Unrelated serum (1:250). Secondary anti-α-FITC IgY antibodies (1:1000). Nuclear staining with 4′,6-diamidino-2-phenylindole (DAPI, 1 μg/ml). The images were taken at ×100 magnification using a Nikon C1 plus confocal fluorescence microscope

The anti-His-PfNMNAT IgY antibody identifies PfNMNAT with a cytoplasmic pattern. Pre-immune chicken serum was used as a negative control (Fig. 4). Identification was not homogeneous within the cytoplasm of the parasite since there were aggregation points, which can be due to concentration of the protein in specific points or organelles.

**Fig. 5:**
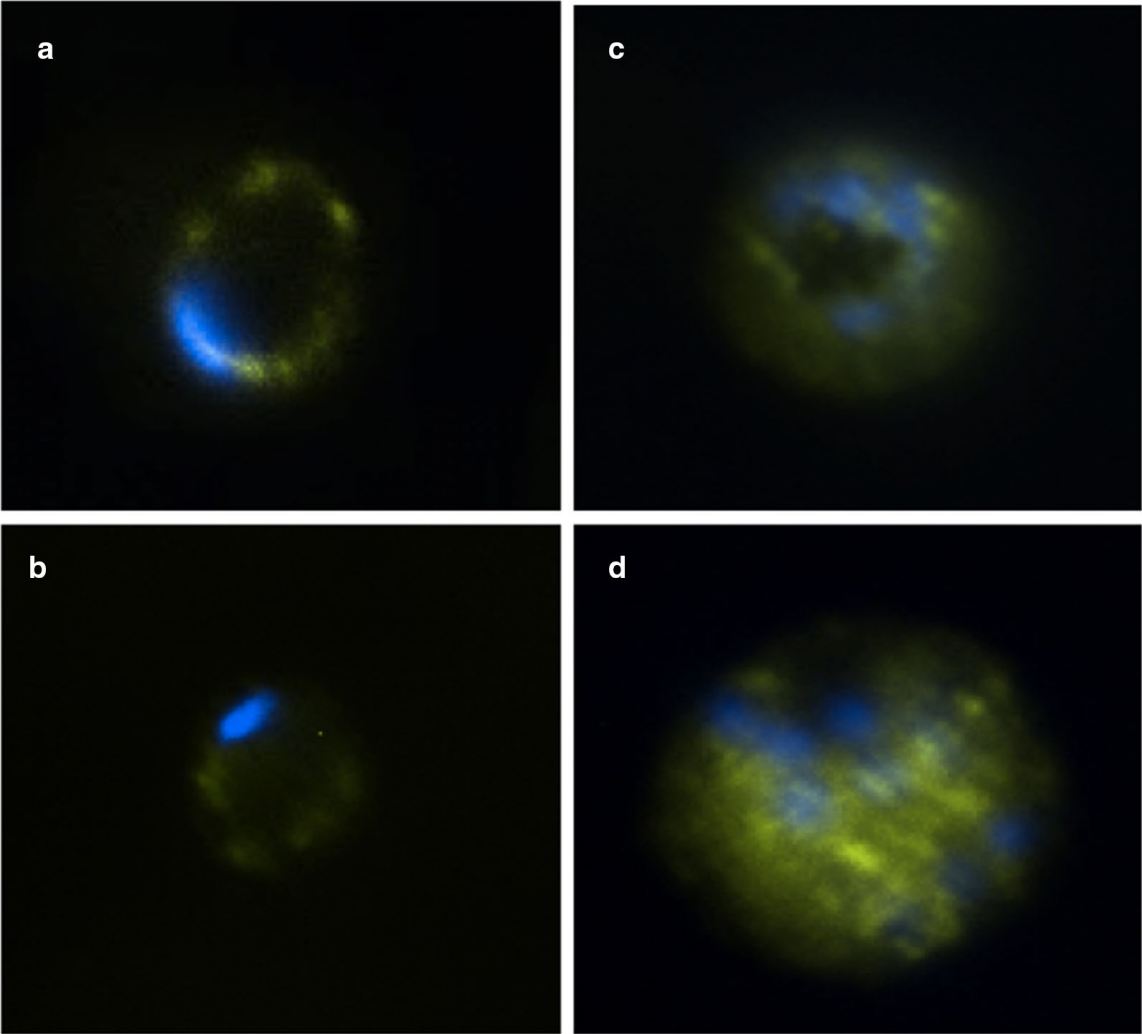
Magnified image of PfNMNAT localization by fluorescence microscopy using IgY antibody. Recognition of the PfNMNAT protein in the FCR3 strain of *P. falciparum* in the stages of rings (**a**), trophozoite (**b**) and schizonts (**c**, **d**). Primary anti-His-PfNMNAT IgY antibody (1:250); secondary anti-α-FITC IgY antibody (1:1000); nuclear staining with DAPI (1 μg/ml)

The use of anti-His-PfNMNAT IgY antibodies specifically showed the protein in the cytoplasm in all three stages of the asexual life cycle (Fig. 5). These results of the immunodetection and immunolocalization of the endogenous protein confirm the localization described by O’Hara et al. where the overexpression of the GFP-coupled recombinant protein in the carboxy-terminal region shows a cytoplasmic profile as well as a possible punctuated pattern of cytoplasmic accumulation [12]. The same localization has been reported for other NMNATs, e.g., mouse [25], yeast [26] and for one of the NMNAT isoforms of *Drosophila melanogaster* [27]. The NAD+ synthesized in this compartment can be used in cytoplasmic processes, such as in mobilizing calcium to the endoplasmic reticulum as precursor molecules of agents, in metabolic pathways such as glycolysis [28], and in the synthesis of calcium-mobilizing agents such as cyclic adenosine diphosphate ribose (cADPR) and nicotinic acid adenine dinucleotide phosphate (NAADP) [29]. The fact that the parasite only has one NMNAT, could be attributed a simplified metabolic pathways in the parasite, as well as intracellular parasites such as *Leishmania braziliensis* and *Trypanosoma cruzi* in comparison with free-living organisms [9, 10]. In this way, NAD+ can be transported to the nucleus and mitochondria [30, 31] by a still unknown mechanism.

### PfNMNAT expression varies during the asexual life cycle

**Fig. 6:**
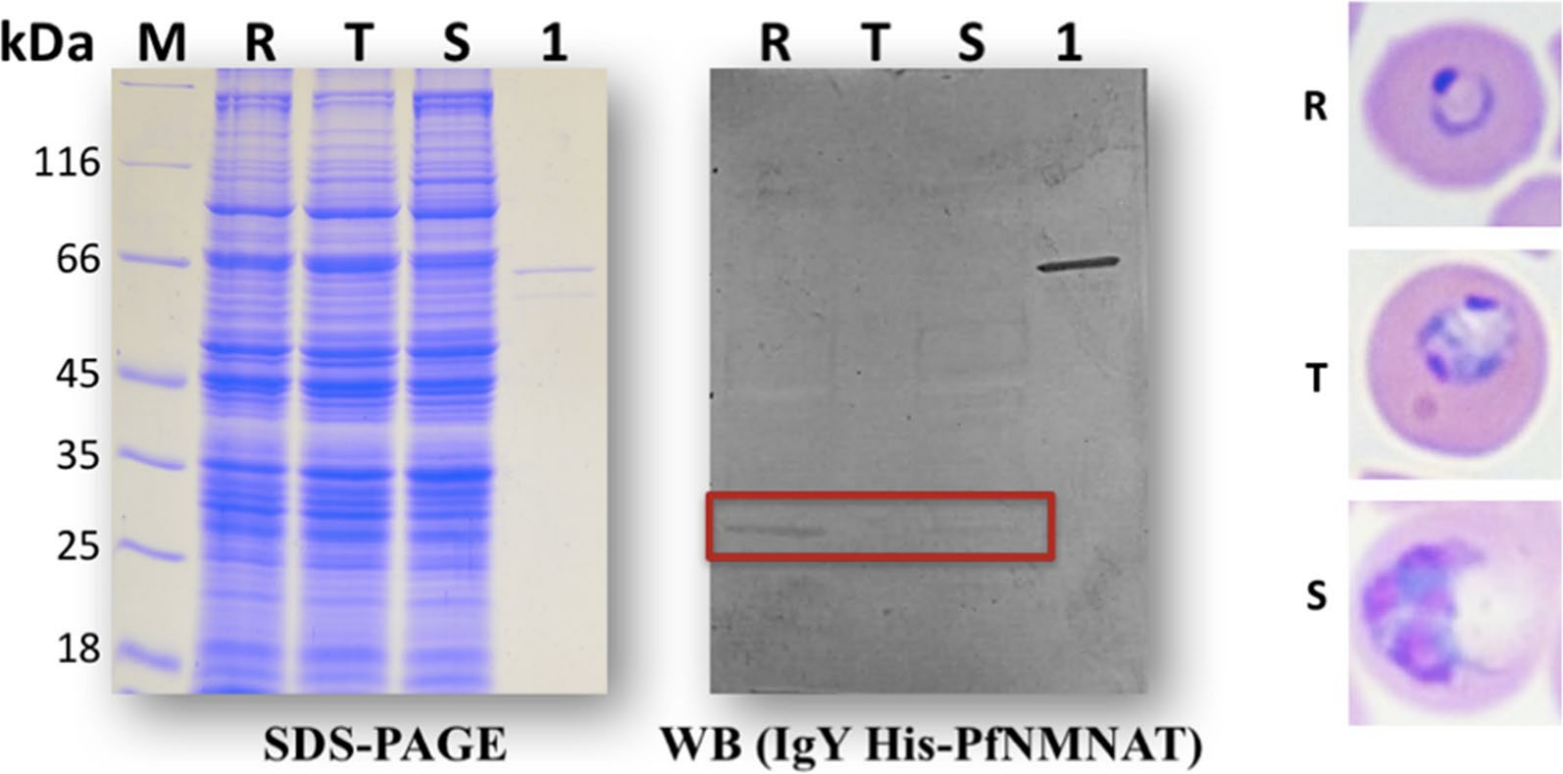
Protein expression analysis of PfNMNAT. 150 μg protein from the ring (R), trophozoite (T) and schizont (S) stages were loaded. As a positive control, 150 ng of recombinant protein MBP-PfNMNAT (66 kDa) was loaded (1). M. molecular weight marker (Thermo Scientific). 12% SDS-PAGE stained with Coomassie Blue R-250 and western blot on a PVDF membrane. Primary anti-His-PfNMNAT IgY antibody (1:1000). Secondary antibody: biotinylated anti-chicken (1:8000). Streptavidin–alkaline phosphatase (1:8000). Developed with the BCIP/NBT substrate system

Western blot analysis of protein extracts using anti-His-PfNMNAT IgY serum, allowed identification that the PfNMNAT protein is expressed mostly in the ring (Fig. 6), being a result consistent with what is reported in the database of *Plasmodium* [32] whereby ribosomal profile, it was found that in the ring stage the PfNMNAT is translationally more active in comparison with the other stages of the asexual life cycle [33].

The results suggest that the regulation of PfNMNAT expression may be occurring at the post-transcriptional level, as has been shown for the parasite in recent studies [34, 35], because the overall analysis of transcripts for *Plasmodium* revealed that NMNAT does not have significant variations in the asexual life cycle [36]. The parasite genome does not code for specialized transcription factors, whereas there are many genes coding for binding proteins and RNA regulation, indicating a possible regulation at levels subsequent to transcription [37–39].

## Conclusion

PfNMNAT was immunodetected using different antibodies, observing a higher molecular weight band than expected, which could be attributed to post-translational modification. The cytoplasmic intracellular localization of the NMNAT protein in *P. falciparum* parasites was proven in the three stages of the asexual life cycle. PfNMNAT was observed at the transcriptional and protein levels. Protein levels showed variations during the asexual life cycle, with one major expression peak in the ring stage. The results of this work suggest that PfNMNAT is being regulated at post-transcription levels.

## Additional files

**Additional file 1.** Evaluation of anti-His-PfNMNAT IgY (Serum) antibodies on the recombinant protein.

**Additional file 2.** Evaluation of anti-His-PfNMNAT IgY (Yolk) antibodies on the recombinant protein.
