# Supplementary material for: Localization and phosphorylation of Plasmodium falciparum nicotinamide/nicotinate mononucleotide adenylyltransferase (PfNMNAT) in intraerythrocytic stages
Source: Malar J. 2018 Apr 11;17:161. doi: 10.1186/s12936-018-2307-4 (PMC5896089; doi:10.1186/s12936-018-2307-4)
Supplement: Supplementary file 2 — Additional file 2. Evaluation of anti-His-PfNMNAT IgY (Yolk) antibodies on the recombinant protein. [file 12936_2018_2307_MOESM2_ESM.docx]

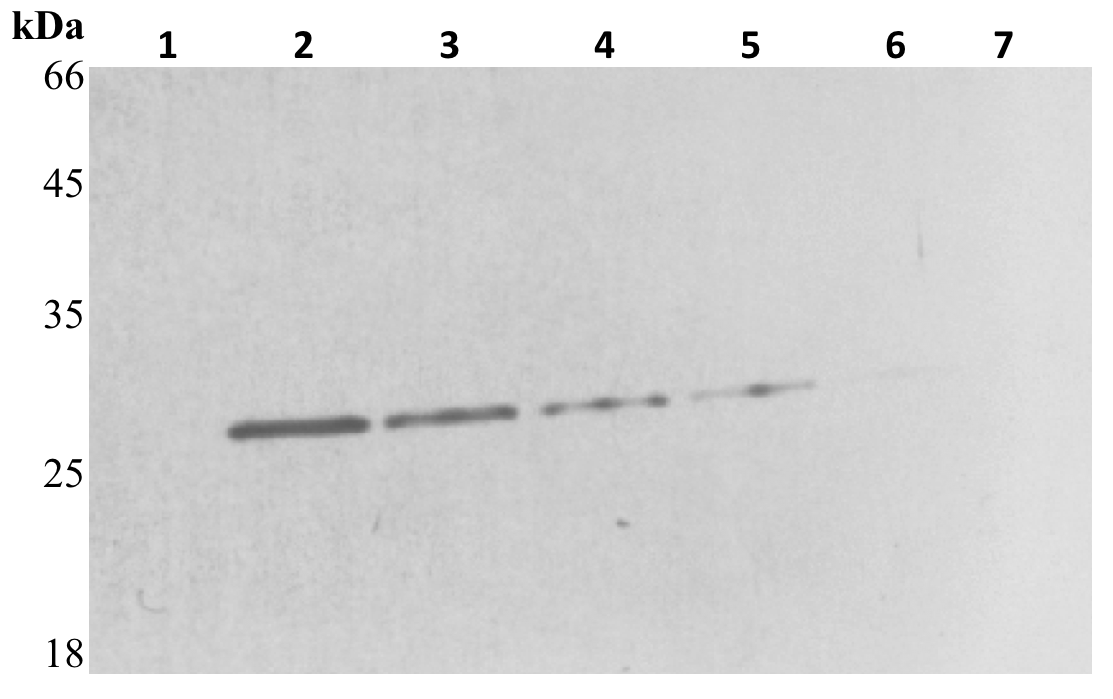


**Additional file 2 Evaluation of anti-His-PfNMNAT IgY (Yolk) antibodies on the recombinant protein**

Western blot, PVDF membrane, HRP system. IgY primary antibody (1:5000) for identification of recombinant His-PfNMNAT: 1. 125 ng BSA; 2-7. His-PfNMNAT: 2. 125 ng; 3. 60 ng; 4. 30 ng; 5. 15 ng; 6. 7 ng; 7. 3 ng.
